# Intelligent Metal-Phenolic Metallogels as Dressings for Infected Wounds

**DOI:** 10.1038/s41598-019-47978-9

**Published:** 2019-08-09

**Authors:** Ha Thi Phuong Anh, Chun-Ming Huang, Chun-Jen Huang

**Affiliations:** 1 Department of Biomedical Sciences and Engineering, National Central University, Jhong-Li, Taoyuan 320 Taiwan; 2 Department of Chemical and Materials Engineering, National Central University, Jhong-Li, Taoyuan 320 Taiwan; 3 R&D Center for Membrane Technology, Chung Yuan Christian University, 200 Chung Pei Rd., Chung-Li City, 32023 Taiwan

**Keywords:** Bioinspired materials, Chemical engineering

## Abstract

In this study, we report a metallogel developed based on metal-phenolic coordination of natural low-cost polyphenolic molecule and metal ions. Gelation occurs by mixing tannic acid (TA) and group (IV) titanium ions (Ti^IV^) to form TA-Ti^IV^ gel. The TA-Ti^IV^ gel exhibits good capability to incorporate diverse metal ions by *in situ* co-gelation. Herein, five antimicrobial metal ions, i.e. ferric (Fe^III^), copper (Cu^II^), zinc (Zn^II^), cobalt (Co^II^) and nickel (Ni^II^) ions, were employed to include in TA-Ti^IV^ gels for developing intelligent dressings for infected wounds. The chemical and coordinative structures of TA-Ti^IV^ metallogels were characterized by UV-Vis and Fourier-transform infrared (FT-IR) spectroscopies. Cytotoxicity of antimicrobial metallogels was explored by MTT assay with NIH 3T3 fibroblasts. The release of metal ions was evaluated by inductively coupled plasma mass spectrometry (ICP-MS), indicating the different releasing profiles upon the coordinative interactions of metal ions with TA. The formation and disassembly of metallogels are sensitive to the presence of acid and an oxidizer, H_2_O_2_, which are substances spontaneously generated in infected wounds due to the metabolic activity of bacteria and the intrinsic immune response. The Cu^II^ releasing rates of TA-Ti^IV^-Cu^II^ metallogels at different pH values of 5.5, 7.4 and 8.5 have been studied. In addition, addition of H_2_O_2_ trigger fast release of Cu^II^ as a result of oxidation of galloyl groups in TA. Consequently, the antimicrobial potency of TA-Ti^IV^-Cu^II^ metallogels can be simultaneously activated while the wounds are infected and healing. The antimicrobial property of metallogels against Gram-negative *Escherichia coli*, and Gram-positive *Methicillin-Resistant Staphylococcus aureus* (USA300) and *Staphylococcus epidermidis* has been investigated by agar diffusion test. In an animal model, the TA-Ti^IV^-Cu^II^ metallogels were applied as dressings for infected wounds, indicating faster recovery in the wound area and extremely lower amount of bacteria around the wounds, compared to TA-Ti^IV^ gels and gauze. Accordingly, the intelligent nature derived metallogels is a promising and potential materials for medical applications.

## Introduction

Infections occur when virulence factors expressed by one or more microorganisms in a wound outcompete the host immune system. Dissemination of microorganisms in tissue provokes a series of local and systemic host responses^1^. Complications of infected wounds depend on the pathogenicity of the microorganisms and on the immune competence of the host, manifesting as clinical signs, such as pain, heat, erythema, edema, tenderness, cellulitis and abscess. The infection in a wound can lead to an acute responses and delay the wound healing process^2^. Bacteria such as methicillin-resistant *Staphylococcus aureus* (USA300), *Escherichia coli* and *Staphylococcus epidermidis* have been frequently isolated from infected wounds, and become common wound pathogens^3^.

It has been proven that the pH of a wound environment plays an important role in wound healing because it relates to antimicrobial activity and control infection, angiogenesis, oxygen release, and protease activity^4^. Bacterial infection leads to the pH fluctuation because of the complex process of healing, immune responses and bacterial metabolism. The pH has been found to fall to the value as low as 5.5 at the infection sites due to low-oxygen fermentation, triggering the production of lactic acid and acetic acid^5–7^. In addition, the production of hydrogen peroxide (H_2_O_2_) in the infecting wounds is the first response of the immune system during inflammation to exert antimicrobial activity^8,9^. H_2_O_2_ is known as a reactive oxygen species (ROS), which accounts for their versatility in mediating host defense against a broad range of pathogens. In addition, studies revealed that H_2_O_2_ supports the healing process at a level of µM concentration by serving as a signaling molecule and stimulating an effector cells response^10,11^. The endogenous substances allow to serve as an environmental stimulus to spontaneously trigger the antimicrobial reactions of medical devices. Therefore, pH- and H_2_O_2_-responsive antibiotic delivery systems have received considerable attention due to their great potential and easy applicability in the clinical treatment of infections^5,12,13^.

Antimicrobial properties of metal ions have been known and explored for many years^14^. Antibacterial metal ions, such as silver (Ag^I^), zinc (Zn^II^), cobalt (Co^II^), nickel (Ni^II^), iron (Fe^III^) and copper (Cu^II^), have been extensively used in artificial medical implants and devices due to their broad-spectrum antibacterial activity^14–16^. The toxicity of metals to bacteria is primarily related to strong affinities to biomolecules and production of ROS. Recent studies indicate that different metals cause discrete and distinct types of injuries to microbial cells as a result of protein dysfunction, oxidative stress or membrane damage^17^. Generally, metals appear to target multiple cellular processes, leading to pleiotropic effects on bacteria^17^.

Polyphenolic derivatives exist ubiquitously in nature. Their coordinative complexation with metal ions displays a remarkable degree of physico-chemical versatility that has already inspired an ever-growing number of applications as functional materials^18–21^. Harrington *et al*. indicated that catecholic amino acid-iron chelate complexes form the peculiar metallopolymeric structures, exhibiting a combination of high hardness and high extensibility^21^. Recently, Caruso’s group demonstrated metal-phenolic complexation between different metals and natural polyphenolic tannic acid (TA) for assembly of conformal functional coatings^20^. Moreover, they reported a formation of metal-phenolic supramolecular metallogels by direct complexation of multitopic galloyl groups of TA and group (IV) transition metal of Ti^IV^ ^14^. The metallogels represent excellent versatility to incorporate diverse metal ions, nanomaterials, and polymers via *in situ* co-gelation without affecting the fundamental gelation process. Additionally, the metallogels display pH-modulated mechanical property, shape persistence, adhesiveness, optical transparency, tunable mechanical properties and self-healing capability^14,20^.

In this study, we attempted to develop antibacterial metallogels with pH- and H_2_O_2_-responsive properties as dressings for infected wounds. The metallogels were synthesized via co-gelation of TA, high-oxidation state Ti^IV^ and antimicrobial metal ions for formation of three-dimensional metal-phenolic networks (MPNs). The antimicrobial metal ions of Zn^II^, Co^II^, Ni^II^, Fe^III^ and Cu^II^ were included in the metallogels for comparative study in terms of their release profiles, cytotoxicity, and antimicrobial potency. Moreover, the antibacterial effect of metallogels can be triggered by acids originated from the metabolic activity of bacteria, and endogenously produced H_2_O_2_ after skin wound occurs upon disassembly of the MPNs. UV-vis spectroscopy and Fourier-transform infrared spectroscopy (FTIR) were employed for analysis of coordinative structures of metallogels. The metal ion release profiles and responsiveness of the metallogels to pH and H_2_O_2_ were determined by inductively coupled plasma mass spectrometry (ICP-MS). The antimicrobial properties and cytotoxicity of all metal ions were accessed by minimum inhibitory concentration (MIC) test and 3-(4,5-dimethylthiazol-2-yl)-2,5-diphenyl tetrazolium bromide (MTT) assay. The antimicrobial effect of metallogels against Gram-negative *E. coli*, and Gram-positive *S. aureus* and *S. epidermidis* was investigated by agar diffusion test. In an animal model, biocompatible TA-Ti^IV^-Cu^II^ metallogels were developed as dressings for infected wounds on mice in comparison with TA-Ti^IV^ gels and gauze. Ultimately, the versatility and robustness, combined with the low cost, biocompatibility, ease, and scalability of the coordination-driven assembly make the metallogels excellent candidates for medical applications.

## Experimental Section

### Materials

Tannic acid (TA, ACS reagent), titanium (IV) bis (ammonium lactato) dihydroxide (Ti-BALDH) solutions 50 wt% in H_2_O, iron (III) hexahydrate (FeCl_3_.6H_2_O), copper (II) chloride (CuCl_2_), cobalt (II) bromide (CoBr_2_), nickel (II) chloride (NiCl_2_), zinc (II) chloride (ZnCl_2_), 2,6-dichlorophenolindophenol, trizma hydrochloride (Tris), copper assay kit and peroxidase from horseradish (HRP) lyophilized powder were purchased from Sigma-Aldrich. Phosphate buffer saline (PBS) was obtained from Acros Organics. Luria-Bertani (LB) and tryptic soy broth (TSB) were purchased from Gibco. MTT assay kit and sodium hydroxide (NaOH) were acquired from Thermo Fisher Scientific. Dulbecco’s Modified Eagle’s Medium (DMEM) and fetal bovine serum (FBS) were purchased from Gibco. NIH 3T3 fibroblasts were obtained from Bioresource Collections and Research Center (BCRC), Taiwan. *S. epidermidis* ATCC12228, methicillin-resistant *S. aureus* (USA300, ATCC BAA-1718) and *E. coli* ATCC12435 were acquired from Bioresource Collections and Research Center in Taiwan. All the water used was purified to 18.2 mΩ using a Millipore water purifications system, and filtered using a 0.22 µm filter.

### Preparation of antimicrobial metallogels

All solutions were prepared under a nitrogen atmosphere using standard Schlenk techniques. The preparation of TA-Ti^IV^ metallogels was referred to previous study^14^. Briefly, 5 wt% of TA solution was prepared in deionized water. The pH of the TA solution was adjusted to approximately 7 by adding 1 M NaOH solution. 50 wt.% of Ti-BALDH stock solutions was then mixed with the TA solutions to afford a TA:Ti molar ratio of 1:5. The mixture was thoroughly shaken for 10 s to make sure all the components were well mixed and homogeneous. The mixed solution was left to stand for 20 min and the color of the solution turned from orange to red. The state of the solution was turned from sol to gel. The hydrogel was removed from the tube and denoted as TA-Ti^IV^.

The antimicrobial metal ions of Fe^III^, Cu^II^, Co^II^, Ni^II^ and Zn^II^ were introduced individually for preparation of the metallogels by co-gelation. The Ti^IV^ and antimicrobial metal ions were added into the TA solution, prepared as above mentioned. The mixed solution had a molar ratio of TA:Ti^IV^:M to be 1:4:1, where M is referred to the antimicrobial metal ions. The mixture was shaken with vortex for 10 s and then stood on a bench for 20 min.

### Spectroscopic characterizations of metallogels

UV-vis spectroscopy was used to examine the coordination reaction of TA and Ti^IV^ in an aqueous solution. UV–vis spectra were obtained on a spectrophotometer (V-600, JASCO, MD) at room temperature. Three solutions were prepared prior to the measurements. Control samples of TA and Ti^IV^ solutions were prepared in oxygen-free deionized water at concentrations of 0.1 and 0.3455 wt%, respectively. The pH of the TA solution was controlled at 7. The TA-Ti^IV^ solution was prepared as above mentioned.

Fourier-transform infrared spectroscopy (FTIR, Jasco FT/IR-410) was employed to analyze chemical and coordination structures of metallogels. TA-Ti^IV^ metallogels were dried in liquid nitrogen under reduced pressure for 4 h. The dried TA-Ti^IV^ was grounded by a pestle and blended with KBr. The mixed powder was compressed into a pellet. FTIR was operated in a wavenumber range of 400 to 4000 cm^−1^.

### Release profiles of metal ions

Metal ion release profile was measured and recorded for 15 h using inductively coupled plasma-mass spectrometer (ICP-MS, Agilent 7500ce, Japan). The metal-contained metallogels were cut into round discs with a diameter of 15 mm and thickness of 1.5 mm. The metallogel disc was immersed into 5 mL of PBS buffer at pH of 7.4 at 37 °C with agitation at 30 rpm. At each time point, the solution was collected for measuring the accumulative release of metal ions. 5 mL of fresh PBS buffer has added to compensate for replacing solutions.

### pH- and H_2_O_2_-responsiveness of metallogels

The pH- and H_2_O_2_-responsiveness of TA-Ti^IV^-Cu^II^ metallogels were characterized by measuring the amount of released Cu^II^. The released Cu^II^ was quantified at incubation time points of 12, 24 and 36 h by ICP-MASS. The TA-Ti^IV^-Cu^II^ metallogels were cut into round discs with a diameter of 15 mm and thickness of 1.5 mm. For the pH-responsiveness, the metallogels, buffered solutions at pH values of 5.5, 7.4 and 8.4 were prepared. The TA-Ti^IV^-Cu^II^ metallogels were immersed in 5 mL of buffered solutions at 37 °C with agitation at 30 rpm. At every 12-h period of time, the concentration of Cu^II^ in the solution was measured using the assay kit. Afterward, fresh 5 mL of the buffered solution was added for the next measurement.

For the H_2_O_2_ responsiveness test, the concentration of Cu^II^ in ppm, released from TA-Ti^IV^-Cu^II^ metallogels, was determined after incubation with H_2_O_2_ in a buffer at pH 5.5 at 37 °C for 36 h. To further confirm the H_2_O_2_ responsiveness of metallogels, HRP, that catalyzes the oxidation of various organic substrates by H_2_O_2_, was included in the metallogels to observe the release of Cu^II^. For the HRP encapsulation, HRP in PBS was introduced to mix with the TA solution to achieve the final concentration in each gel is 62.5 U/gel and 31.5 U/gel. The size of the metallogels was described as above, which is a round disc with a diameter of 15 mm and thickness of 1.5 mm. After mixture, Ti^IV^ and Cu^II^ were added and vortexed for 10 s. The metallogels containing HRP (TA-Ti^IV^-Cu^II^-HRP) were formed after 20 min. The test for the release of Cu^II^ in the presence of H_2_O_2_ was determined at pH 5.5 at 37 °C for 36 h.

### Minimum inhibitory concentrations (MICs) of metal ions

MIC is the concentrations at which an antibacterial agent experiences the complete inhibitions of the growth of microorganisms^22^. The MICs of metal ions were determined using the microbroth dilution method in a 96-well plate for the three bacteria (*E. coli*, *S. aureus* and *S. epidermidis*). *E. coli* was grown overnight in LB media, and *S. aureus* and *S. epidermidis* were grown overnight in TSB media. The bacterial solutions were diluted to an optical density of 0.1 at 670 nm (OD670) in corresponding medium. Consequently, 100 µL of the bacterial solutions were distributed to each well of the 96-well plate. The concentrations of metal ions were prepared, ranging from 0.125 µM to 32 µM. The bacterial solutions in wells were incubated with metal ions for 10 h, followed by measuring the OD670 values of the testing solutions by microplate reader (Synergy 2 Multi-Mode, BioTek, USA). The growth curves of bacteria in metal ion solutions were shown in Supplementary Information (Figs S1–S3).

### Cytotoxicity of metal ions

The cytotoxicity of metal ions for NIH 3T3 fibroblast cells was evaluated by MTT assay. The concentrations of metal ion solutions were prepared ranging from 31.25 µM to 250 µM. 3T3 fibroblasts cells were cultured in a 24-well plate at a seeding concentration of 1 × 10^4^ cell/well in DMEM containing 10% of FBS in a humidified 5% CO_2_ incubator at 37 °C for 24 h. Afterward, the culture medium was replaced by FBS-free DMEM, which contained metal ions of interest at various concentrations. After incubation for 24 h, the MTT solution was introduced to the culture medium to a final concentration of 0.5 mg/mL. MTT, a yellow tetrazole, is reduced to purple formazan in living cells. Therefore, after incubations for 3 h, the medium was replaced by DMSO in each well to dissolve the purple formazan crystals. The absorbance of solutions was quantified by a spectroscopic reader at 550 nm. The cell culture without treatment was set as a control. The resulting value was averaged from triplicate samples.

### Tests for zone of inhibition

Antibacterial activity of metallogels was accessed by disc diffusion test by measurement of zone of inhibition. *E. coli* was cultured in sterile LB liquid culture medium; *S. epidermidis* and USA300 were incubated in sterile TSB at 37 °C for 24 h. Then, the bacterial solutions were diluted to 0.1 of OD670 using the corresponding medium, followed by dropping 10 µL of diluted bacterial solutions onto sterile agar plates and spreading homogeneously. Six samples of metallogels, TA-Ti^IV^, TA-Ti^IV^-Fe^III^, TA-Ti^IV^-Cu^II^, TA-Ti^IV^-Co^II^, TA-Ti^IV^-Ni^II^, and TA-Ti^IV^-Zn^II^ were cut into a round shape with a diameter of 8 mm and thickness of 1.5 mm. The samples were placed on the agar plates in an incubator for 24 h. The zone of inhibition is measured using a pair of calipers. Its size is rounded off to the closest millimeter, and the diameter of the disk is also included.

### Animal tests

This study was carried out in strict accordance with an approved Institutional Animal Care and Use Committee (IACUC) protocol at National Central University (NCU), Taiwan. The experimental animals and were purchased from BioLASCO Taiwan Co., Ltd. The animal experiments were conducted referring to previous work^23^. Six female mice weighed 250–300 g were used in this study. Animals were anesthetized via injection of 2,2,2-tribromoethanol. Dorsal skin was shaved and sanitized. A circular full-thickness wound was created by a punch with a 6 mm diameter. 10 µL of pathogenic *S. aureus* bacterial solution at a concentration of 10^7^ CFU/mL in PBS was dropped on the wounds. Three dressings, including gauze, TA-Ti^IV^ and TA-Ti^IV^-Cu^II^ metallogels were placed on the wounds, followed by covering Tegaderm (3 M, USA) to immobilize the dressings. Dressings were changed on day 1, day 4, and day 6. The appearance of wounds was observed and photographed. The analytical equations of the percentage of the wound area were based on the ratio of recovered wound area and created wound area on day 0. The animal experiments completed when wounds with one type of dressing were apparently healed. Animals were euthanized at the endpoint of day 8. In addition, the concentrations of *S. aureus* on infected wounds with treatment of TA-Ti^IV^, and TA-Ti^IV^-Cu^II^ were determined on 8 day. The cotton stick was used to collect bacteria on wounds and put into 1 mL PBS solution, followed by bacterial culture on TSB agar supplemented with 1% methicillin and incubation at 35 °C for 24 h. Afterward, the colony forming unit (CFU) on agar was calculated.

### Statistical analysis

Values are expressed as means ± standard deviation (S.D.) unless otherwise indicated. Student’s t-test was used for comparisons of data to assess significant differences.

## Results and Discussion

### Formation of metallogels

**Figure 1:**
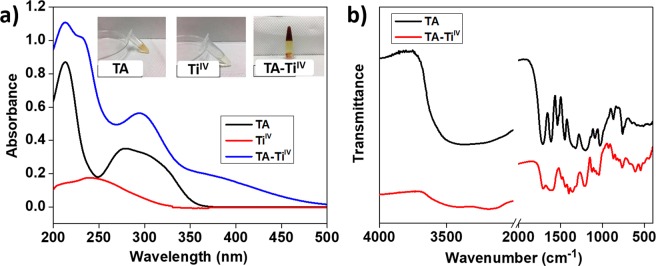
Characterizations of the formation of TA-Ti^IV^ metallogels using UV-vis (**a**) and FTIR (**b**) spectroscopies. The inserted photographs in (**a**) indicate the solutions of TA, Ti^IV^, and TA-Ti^IV^ in tubes.

TA and Ti^IV^ were mixed at pH 7 for the formation of TA-Ti^IV^ metallogels. UV-vis and FTIR spectroscopies were employed to characterize coordinative interactions of the metal ions and phenolic groups. In Fig. 1a, the UV-Vis spectrum of the TA solution presents two absorbance peaks at 213 and 279 nm. The Ti^IV^ solution shows a broad band in the range 225–275 nm with a maximum at 239 nm. After adding the Ti^IV^ solution in the TA solution, an increasing in absorbance in a range of 375–450 nm, corresponding to coordinative interaction. The results are consistent with the previous literature^14^, indicating the formation of bis/tris-type chelating structures^24^. TA provides multiple galloyl chelating sites, which can coordinate with various transition metals. The stable coordination structure between TA and Ti^IV^ can be explained by high oxidation state and formal charge of Ti^IV^, which plays a significant role in the solvent-trapping process of the gelation. Moreover, the inserted photographs in Fig. 1a display appearance of TA, Ti^IV^ solutions and the TA-Ti^IV^ metallogel. The gel was formed after mixing for 15 min and the color of solutions turned to red, reflecting the absorbance at high wavelength from the UV-vis spectroscopic measurements.

Additionally, the interactions between TA and Ti^IV^ in metallogels were investigated by FTIR spectroscopy (Fig. 1b). The FTIR spectrum of TA shows a characteristic peak at 3369 cm^−1^ for stretching vibration of H-bonded –OH^25^, and a peak at 1711 cm^−1^ for stretching vibration of C=O (carboxylic ester) groups^26^, and a peak at 1612 cm^−1^ for aromatic C=C in free phenolic groups^27^. Moreover, peaks at 1535 and 1447 cm^−1^ were attributed to vibrations of aromatic C-C stretching^25^. Peaks at 1320 and 1202 cm^−1^ correspond to O-H deformation of phenolic, and a peak at 758 cm^−1^ was represented for vibration of aromatic C-H^4,16^. The shifts of the characteristic peaks for TA-Ti^IV^ metallogels with respect to the TA alone have been proven by the disappearance of the H-bonded –OH and manifested by a broadened peak at 3182 cm^−1^ due to the formation the coordinative bonding between –OH and Ti^IV^. Moreover, the peaks for the stretching of aromatic C–C in TA shifted to 1484 and 1439 cm^−1^ due to deprotonations of the hydroxyl groups and complexation with metal ions. In addition, the coordinative interaction attributed to the shifts of the vibrations of O–H bonds to 1400, 1216, and 764 cm^−1^ ^4,25^. Consequently, the formation of metallogels through the chelation of phenolic groups in TA and Ti^IV^ was demonstrated by spectroscopic analysis.

### Release profiles of antimicrobial metal ions from metallogels

**Figure 2:**
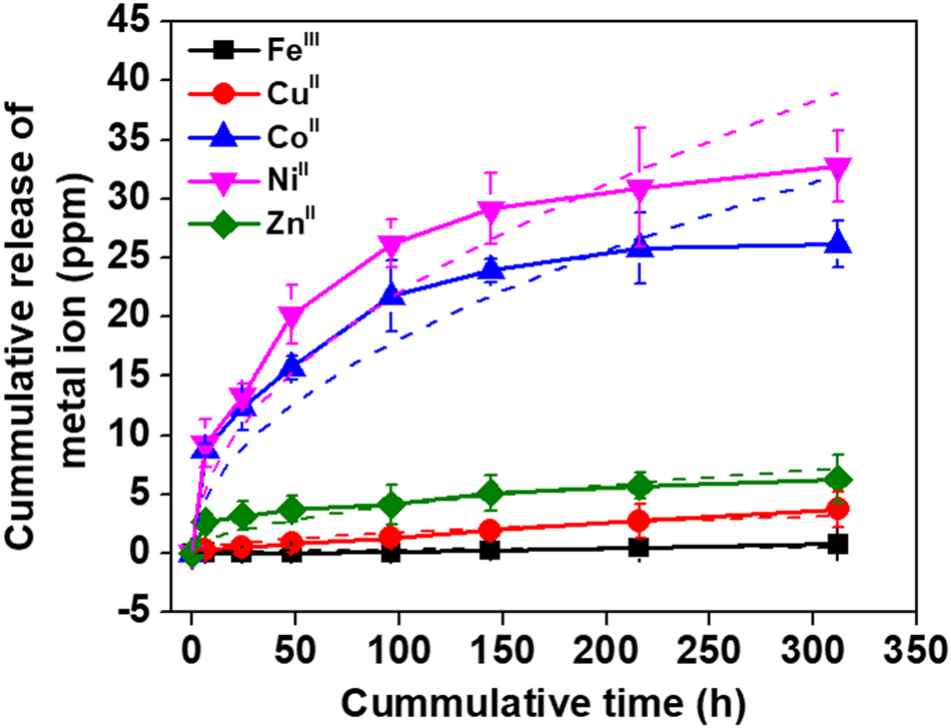
The release profiles of metal ions from metallogels in PBS at pH 7.4 and 37 °C. The cumulative release concentrations of metal ions were recorded using ICP-MASS for 13 days. The data was fitted by using the Higuchi model (dash lines).

Antimicrobial metal ions of Fe^III^, Cu^II^, Co^II^, Ni^II^, and Zn^II^ were incorporated into the metallogels. The release profiles of the metal ions were recorded by using ICP-MASS to determine the difference in the coordination strength with phenolic groups of TA (Fig. 2). The metallogels of TA-Ti^IV^-Fe^III^, TA-Ti^IV^-Cu^II^, TA-Ti^IV^-Co^II^, TA-Ti^IV^-Ni^II^, and TA-Ti^IV^-Zn^II^ were immersed into PBS at pH 7.4 and physiological temperature of 37 °C. As shown in Fig. 2, Ni^II^ was released from TA-Ti^IV^-Ni^II^ fastest, followed by Co^II^, Zn^II^, Cu^II^, and Fe^III^. The release profiles of metals from metallogels were fitted using Higuchi model^28^:

$${\rm{Q}}={K}_{H}\times {t}^{1/2}$$

where Q is cumulative release amount, t is the release time in hour, and K_H_ is the Higuchi dissolution constant. In this work, the K_H_ values for Fe^III^, Cu^II^, Co^II^, Ni^II^, and Zn^II^ were 0.03, 0.18, 1.81, 2.20, and 0.41, respectively. Therefore, the K_H_ values reflect the release order. The different release profiles of metal ions can be explained by the strength of coordinative interaction with phenolic groups. According to empirical hard-soft acid-base theory (HSAB)^17^, acidic phenolic components, such as TA, have a pKa value in the range of 7–9, thus they are easily deprotonated at or below physiological pH. Deprotonated polyphenol ligands behave as hard Lewis bases, giving rise to large metal-binding constants with hard Lewis acids, such as Ti^IV^ and Fe^III^. In contrast, Cu^II^, Zn^II^, Ni^II^ and Co^II^ are moderate Lewis acids and do not bind as strongly as hard Lewis acid to hard oxygen atoms of phenolic groups. Moreover, previous study indicated Cu^II^ has a higher binding constant with phenolic groups than Zn^II^ ^29^, which is consistent with higher Zn^II^ release rate than Cu^II^ from TA-Ti^IV^ metallogels. It is worthwhile to note that undetectable release of Ti^IV^ was found from the ICP-MASS detection, thereby the structure of the metallogels was maintained throughout the testing period.

### Triggered release of metal ions from metallogels

**Figure 3:**
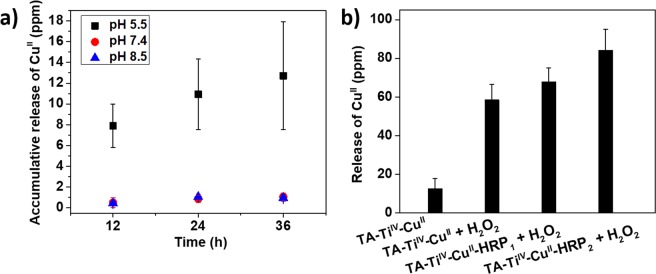
The release of Cu^II^ from metallogels in responses to the changes in pH (**a**) and the presence of H_2_O_2_ at pH 5.5 (**b**).

The susceptibility of the coordinative structures between TA and metal ions to pH and H_2_O_2_ was investigated to explore responsiveness of the metallogels. In Fig. 3a, three different pH values have been used to test the release profiles of Cu^II^ from metallogels of TA-Ti^IV^-Cu^II^. As a result, the release of Cu^II^ at pH 5.5 was much faster than that at higher pH, indicating the disassembly of the phenol-metal coordinative structure^14^. At a low pH value, the galloyl groups of TA are predominately protonated and relatively few phenolate binding sites are available for complexations with Cu^II^, facilitating the high accumulative concentration. On the contrary, at the high pH, the number of deprotonated phenolate groups increases for complexation with Cu^II^.

The release of Cu^II^ from TA-Ti^IV^-Cu^II^ in the presence of oxidizer of H_2_O_2_ was measured in Fig. 3b. The concentration of released Cu^II^ from metallogels in H_2_O_2_ was 4.1-fold higher than that without H_2_O_2_. Moreover, the TA-Ti^IV^-Cu^II^ metallogels were incorporated with HRP enzymes with a concentration of 31 and 62 U/gel, corresponding to TA-Ti^IV^-Cu^II^-HRP_1_ and TA-Ti^IV^-Cu^II^-HRP_2_, respectively. HRP catalyzes the oxidation of various organic substrates by H_2_O_2_. Thus, we suspect that the release of Cu^II^ should be accelerated in the presence of HRP and H_2_O_2_ in the metallogels. The faster release of Cu^II^ from the metallogels associated with the concentration of HRP was observed, and, however, the changes were not dramatic. Therefore, in consideration of cost effectiveness and ease of preparation, the metallgels without HRP were employed for biological tests and applications.

The increased release of Cu^II^ from the metallogels triggered by H_2_O_2_ is ascribed to two mechanisms: 1. the oxidation of phenols to quinones in TA, leading to loss of coordinative capability with Cu^II^ ^30^; 2. the formation of Cu^I^ in the presence of H_2_O_2_ due to the Fenton-like reaction^31^. For the antibacterial property, the redox of Cu^II^ is majorly responsible for killing bacteria through damaging the outer membrane and inhibiting cell respiration by producing a high level of ROS^32^. Cu^II^ was also found to interfere with the replication of 16 S rRNA genes^33^. In addition, Cu^I^ can directly react with certain metabolic enzymes in bacteria and deactivate them^34^. Taken together, the results have further strengthened hypothesis of the enhanced antimicrobial property of TA-Ti^IV^-Cu^II^ in the presence of H^+^ and H_2_O_2_, which endogenously generate in the infected wounds.

### Cytotoxicity test of metallogels

**Figure 4:**
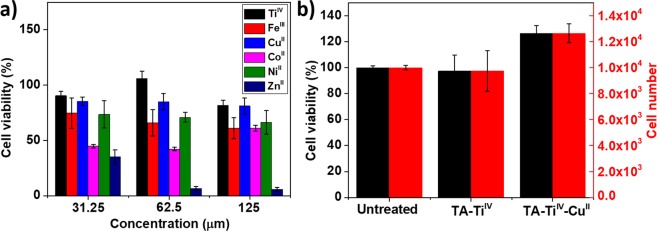
MTT assays for metal ions in solutions (**a**) and metallogels (**b**). NIH-3T3 fibroblasts were used for the MTT assays.

The cytotoxicity of metal ions in solutions was investigated with NIH-3T3 fibroblasts. As shown in Fig. 4a, the concentrations of metal ions were prepared in a range from 31.25 to 125 µM and the solutions were incubated with fibroblasts in a serum-free DMEM for 24 h, followed by the MTT assay. As a result, Co^II^ and Zn^II^ displayed high toxicity to fibroblasts, and Ti^IV^ and Cu^II^ exhibited negligible toxicity in the range of the concentrations. Moreover, cytotoxicity of the metallogels of TA-Ti^IV^ and TA-Ti^IV^-Cu^II^ were accessed in Fig. 4b. Obviously, the growth of fibroblasts was not affected by the metallogels, likely due to low cytotoxicity of natural product of TA and slow release of Cu^II^ and Ti^IV^. The high cell viability for all samples reveals the low cytotoxicity of the metallogels for medical applications.

### Antimicrobial properties of metallogels

MIC tests were conducted for evaluating the antimicrobial capability of all metal ions in solutions. Three bacteria, Gram-negative *E. coli*, Gram-positive *S. aureus*, and *S. epidermidis*, which are common cause of infections involving indwelling foreign devices and surgical wound infections, were employed. In Table 1, *E. coli* seems susceptible to metal ions, likely reflecting their different degrees of interaction with cell wall structures. Generally, different metals cause distinct types of damages to microbial cells as a result of oxidative stress, protein dysfunction, membrane damage and genotoxicity. Co^II^ was more toxic to bacteria than others. The antimicrobial capabilities of Fe^III^ and Cu^II^ metal ions were comparable and moderate.

**Table 1:** MIC tests for metal ions with *E. coli*, *S. aureus* and *S. epidermidis*.

MIC (mM)			
	*E. coli*	*S. aureus*	*S. epidermidis*
Ti^IV^	—	—	—
Fe^III^	4 ± 0.5	8 ± 0.5	8 ± 0.4
Cu^II^	4 ± 0.3	8 ± 0.7	8 ± 1.1
Co^II^	0.5 ± 0.2	1 ± 0.3	2 ± 0.2
Ni^II^	2 ± 0.2	4 ± 0.6	4 ± 0.2
Zn^II^	1 ± 0.3	16 ± 2.2	16 ± 1.5

**Figure 5:**
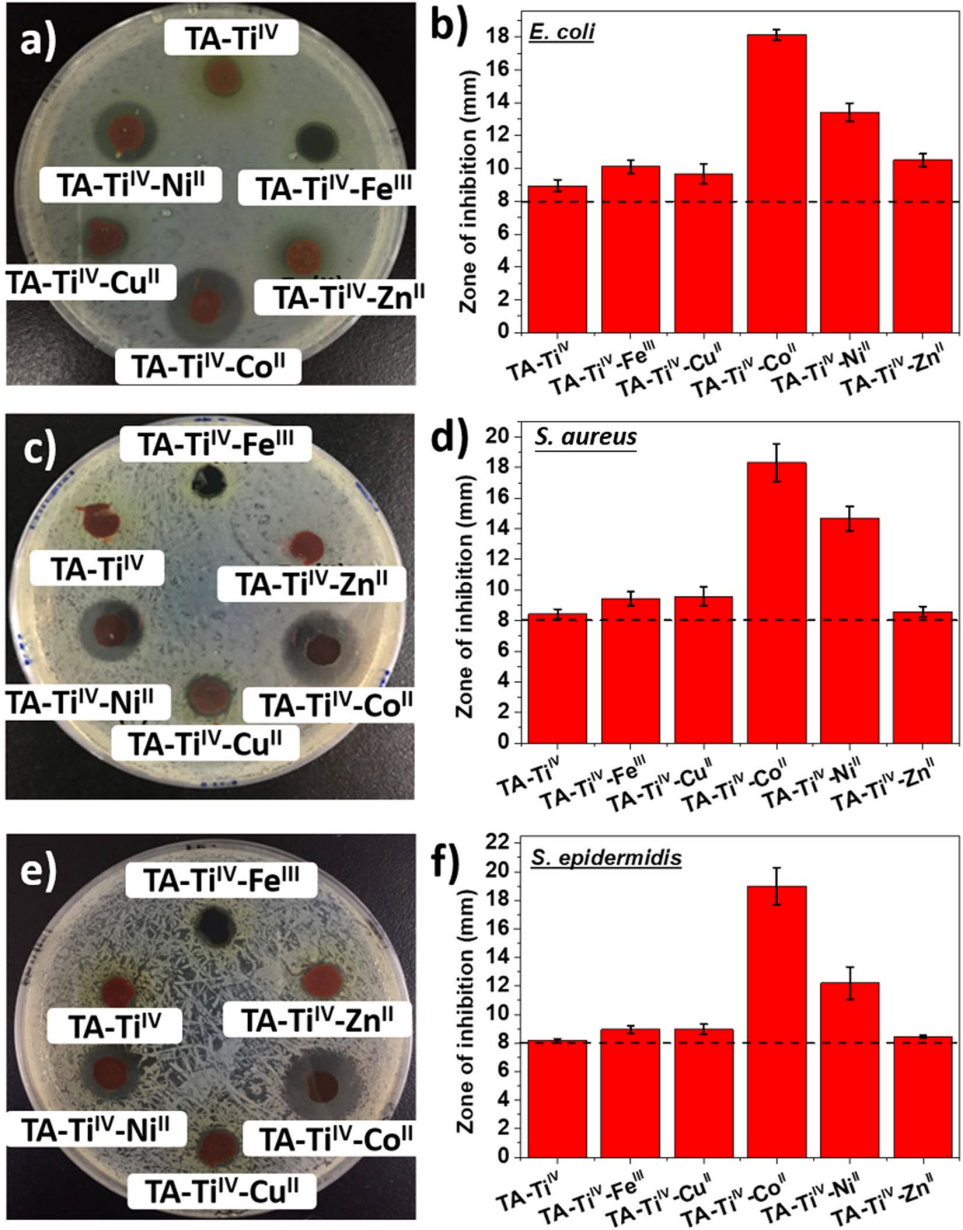
The disc diffusion tests for metallogels of TA-Ti^IV^, TA-Ti^IV^-Fe^III^, TA-Ti^IV^-Cu^II^, TA-Ti^IV^-Co^II^, TA-Ti^IV^-Ni^II^, and TA-Ti^IV^-Zn^II^ with *E. coli* (**a**,**b**), *S. aureus* (**c**,**d**) and *S. epidermidis* (**e**,**f**). The dashed lines indicate the original diameter of the metallogel discs.

The disc diffusion tests were carried out for the metallogels to compare their antimicrobial properties by measuring the diameter of the inhibition zone in Fig. 5. *E. coli* (Fig. 5a,b), *S. aureus* (Fig. 5c,d) and *S. epidermidis* (Fig. 5e,f) were spread and cultured on agar plates, followed by incubation with metallogels for 24 h. From the diameter measurements of Fig. 5b,d and f, the dashed lines indicate the original diameter of the metallogel discs, which is 8 mm. Therefore, TA-Ti^IV^ showed a marginal effectiveness to suppress the growth of bacteria. Moreover, the data from the inhibition zones of TA-Ti^IV^-Zn^II^ are in agreement with that from MIC tests, showing high toxicity to *E. coli*, but not to *S. aureus* and *S. epidermidis*. Moreover, TA-Ti^IV^-Co^II^ exhibited the strongest antimicrobial property against all bacteria, followed by TA-Ti^IV^-Ni^II^. The results can be ascribed to high antimicrobial potency (Table 1) and fast release of Co^II^ and Ni^II^ (Fig. 2) from the metallogels. Although the excellent antimicrobial performance with Co^II^ and Ni^II^ was observed, the high cytotoxicity and fast release from the metallogels constrain medical applications. On the contrary, TA-Ti^IV^-Cu^II^ did not appear to effectively kill bacteria. The good biocompatibility and controlled release of Cu^II^ make TA-Ti^IV^-Cu^II^ a suitable material for medical uses. Consequently, TA-Ti^IV^-Cu^II^ metallogel was employed as a smart dressing for controlled release antimicrobial metal ions in response to the changes in pH and the presence of H_2_O_2_. Herein, the responsiveness of TA-Ti^IV^-Cu^II^ cannot be performed by the disc diffusion test because acid and H_2_O_2_ are bactericidal agents. The test cannot distinguish the antimicrobial roles of metal ions, acid and H_2_O_2_. Accordingly, the antimicrobial and responsiveness of TA-Ti^IV^-Cu^II^ will be demonstrated on infected wounds in mice.

### Metallogels as infected wound dressings

**Figure 6:**
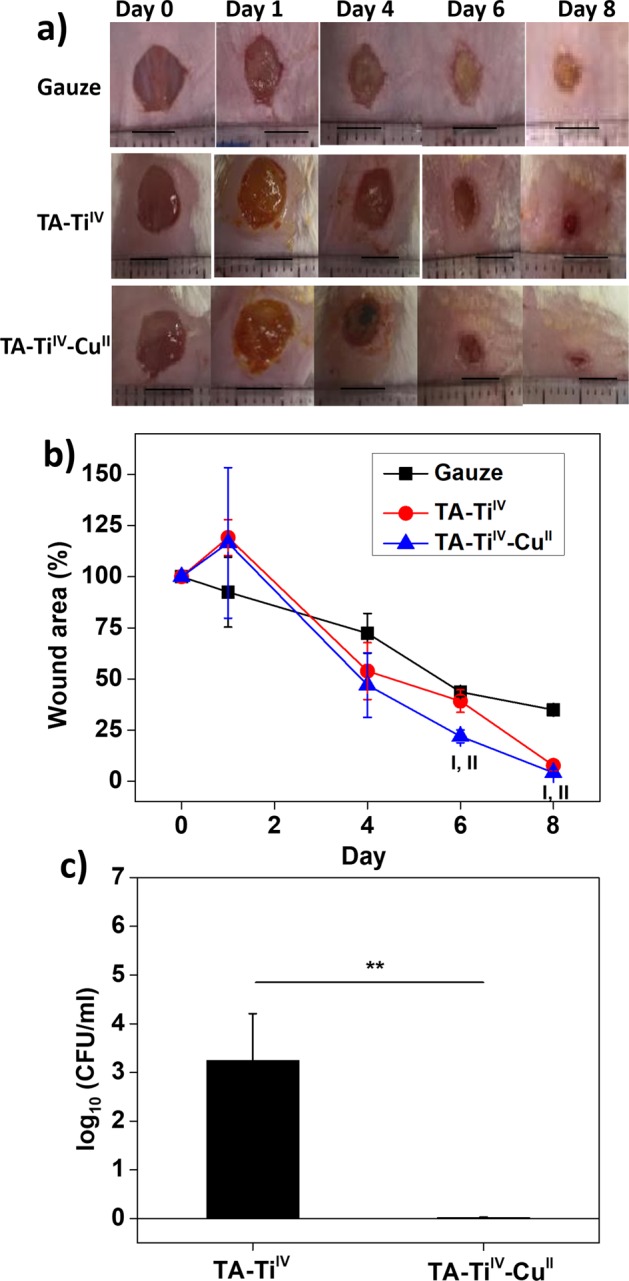
Animal tests for wound dressings for healing of infected wounds. The photographs of wounds with treatment of dressings of gauze, TA-Ti^IV^, and TA-Ti^IV^-Cu^II^ (**a**). Scare bars: 5 mm, n = 3. The wound areas for the wounds with different dressings (**b**). I: significant difference between gauze and TA-Ti^IV^-Cu^II^ (p < 0.05). II: significant difference between TA-Ti^IV^ and TA-Ti^IV^-Cu^II^ (p < 0.05). Bacterial concentrations of infected wounds with treatment of TA-Ti^IV^, and TA-Ti^IV^-Cu^II^ for 8 days (**c**). **p < 0.01.

The use of TA-Ti^IV^-Cu^II^ as a dressing material for infected wounds was conducted to demonstrate the unique characteristics of metallogels. The wounds were created on the back of mice and applied with pathogenic *S. aureus*. Gauze and TA-Ti^IV^ were employed as dressings for comparison. In Fig. 6a, the photographs of wounds with treatment of different dressings were taken periodically to estimate their wound areas. In Fig. 6b, the healing rates of the wounds were determined by reduction in the wound areas. As a result, the wounds with TA-Ti^IV^-Cu^II^ displayed faster recovery than that with other dressings. Moreover, the presence of bacteria on wounds was estimated by counting CFU on tryptic soy broth (TSB) agar supplemented with 1% methicillin. The cotton stick was used to collect bacteria on wounds and put into 1 mL PBS solution, followed by bacterial culture on agar plates to calculate colony forming unit (CFU). In Fig. 6c, obviously, the concentration of bacteria on the wounds with TA-Ti^IV^-Cu^II^ was extremely lower than that with TA-Ti^IV^, showing good antimicrobial potency of TA-Ti^IV^-Cu^II^.

Conclusively, TA-Ti^IV^-Cu^II^ promotes the healing process of the infected wounds due to multiple functions of high hydration, good biocompatibility, and controlled release of antimicrobial agent. Additionally, the gauze adheres to the newly grown tissues on wounds after 3-day use, which occurs very often with traditional dressings when removal of the hydrogel and scab formation^35^. TA-Ti^IV^-Cu^II^ showed very weak adhesion to the wounds due to the hydrophilic surfaces and low cell attachment^36^. Moreover, previous research indicates that Cu^II^ is well-known as an essential element of angiogenesis^37^, and interacts with many factors involved in the wound healing process^38,39^. Therefore, Cu^II^ serves not only an antimicrobial agent, but also a promoter for wound recovery. The responsiveness of the coordinative interaction between galloyl groups and Cu^II^ to the changes of pH and H_2_O_2_ allows spontaneous release of Cu^II^ while the wound is infected. The intelligent property and good biocompatibility of the metallogels enable their implementation in the wound management.

## Conclusions

A nature derived metallogel was successfully fabricated by simple gelation through coordinative interaction between TA and Ti^IV^. Antimicrobial metal ions were incorporated into the metallogels by co-gelation. Because of unique characteristics of phenol-metal chelating interactions, the release of metal ions can be triggered by acid and H_2_O_2_, which are endogenously produced due to the immune response of wounds against bacterial infection. Therefore, the antimicrobial agents spontaneously release while wounds get infected. The cytotoxicity of metal ions and metallogels were carefully evaluated to find a biocompatible dressing material. In this work, TA-Ti^IV^-Cu^II^ metallogel possesses characteristics of controlled release, biocompatibility, pH- and H_2_O_2_-dependent antimicrobial property, and weak adhesion to wounds. We expect that the further development will establish TA-based metallogels as a smart system for advanced biomedical applications.

## Supplementary information

Supplementary Info: Intelligent Metal-Phenolic Metallogels as Dressings for Infected Wounds
