# Supplementary material for: Intelligent Metal-Phenolic Metallogels as Dressings for Infected Wounds
Source: Sci Rep. 2019 Aug 9;9:11562. doi: 10.1038/s41598-019-47978-9 (PMC6688990; doi:10.1038/s41598-019-47978-9)
Supplement: Supplementary file 1 — Supplementary Info: Intelligent Metal-Phenolic Metallogels as Dressings for Infected Wounds [file 41598_2019_47978_MOESM1_ESM.docx]

Supplementary information for Scientific Reports

**Intelligent Metal-Phenolic Metallogels as Dressings for Infected Wounds**

Ha Thi Phuong Anh ^1^, Chun-Ming Huang^1^, Chun-Jen Huang^1,2,3 ,*^

^1^Department of Biomedical Sciences and Engineering, ^2^ Department of Chemical and Materials Engineering, National Central University, Jhong-Li, Taoyuan 320, Taiwan. ^3^R&D Center for Membrane Technology, Chung Yuan Christian University, 200 Chung Pei Rd., Chung-Li City 32023, Taiwan.

*Corresponding author: cjhuang@ncu.edu.tw (C.-J.H.)


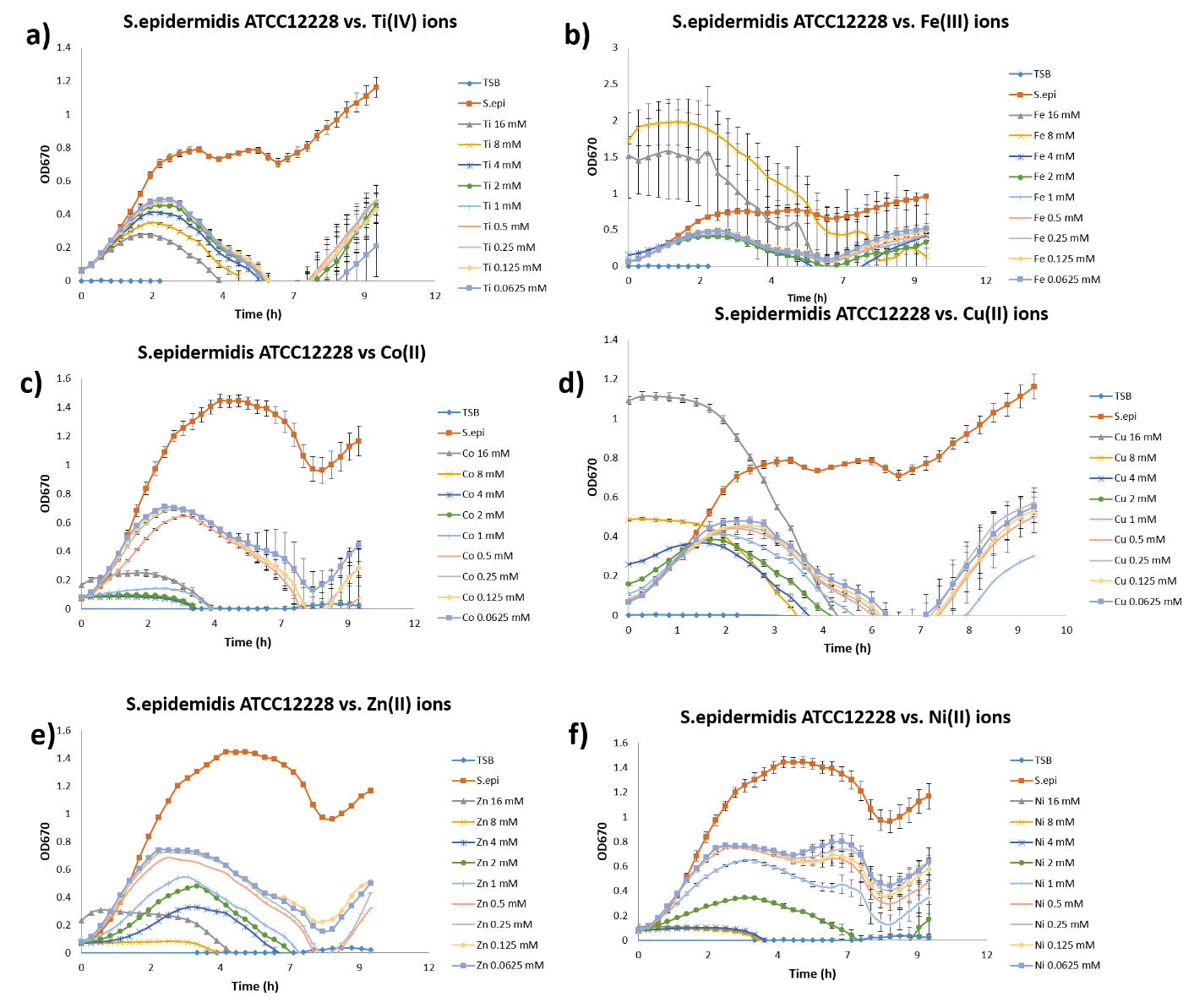


**Figure S1**. The bacterial growth kinetics of *S. epidermidis* with metal ions determined by UV-vis spectroscopy. The bacteria were incubated in metal ion solutions of Ti^IV^ (a), Fe^III^ (b), Co^II^ (c), Cu^II^ (d), Zn^II^ (e) and Ni^II^ (f).


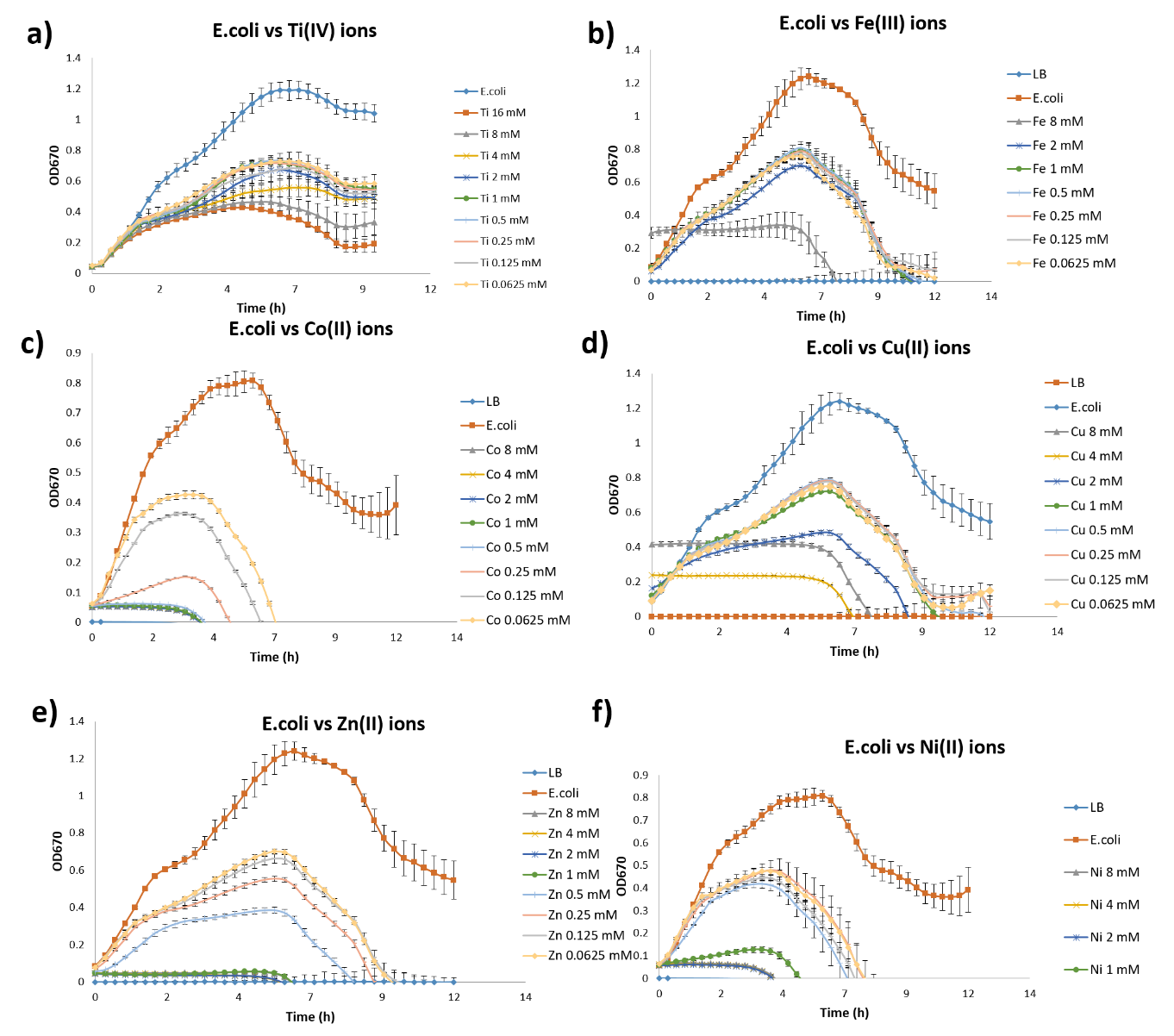


**Figure S2**. The bacterial growth kinetics of *E. coli* with metal ions determined by UV-vis spectroscopy. The bacteria were incubated in metal ion solutions of Ti^IV^ (a), Fe^III^ (b), Co^II^ (c), Cu^II^ (d), Zn^II^ (e) and Ni^II^ (f).


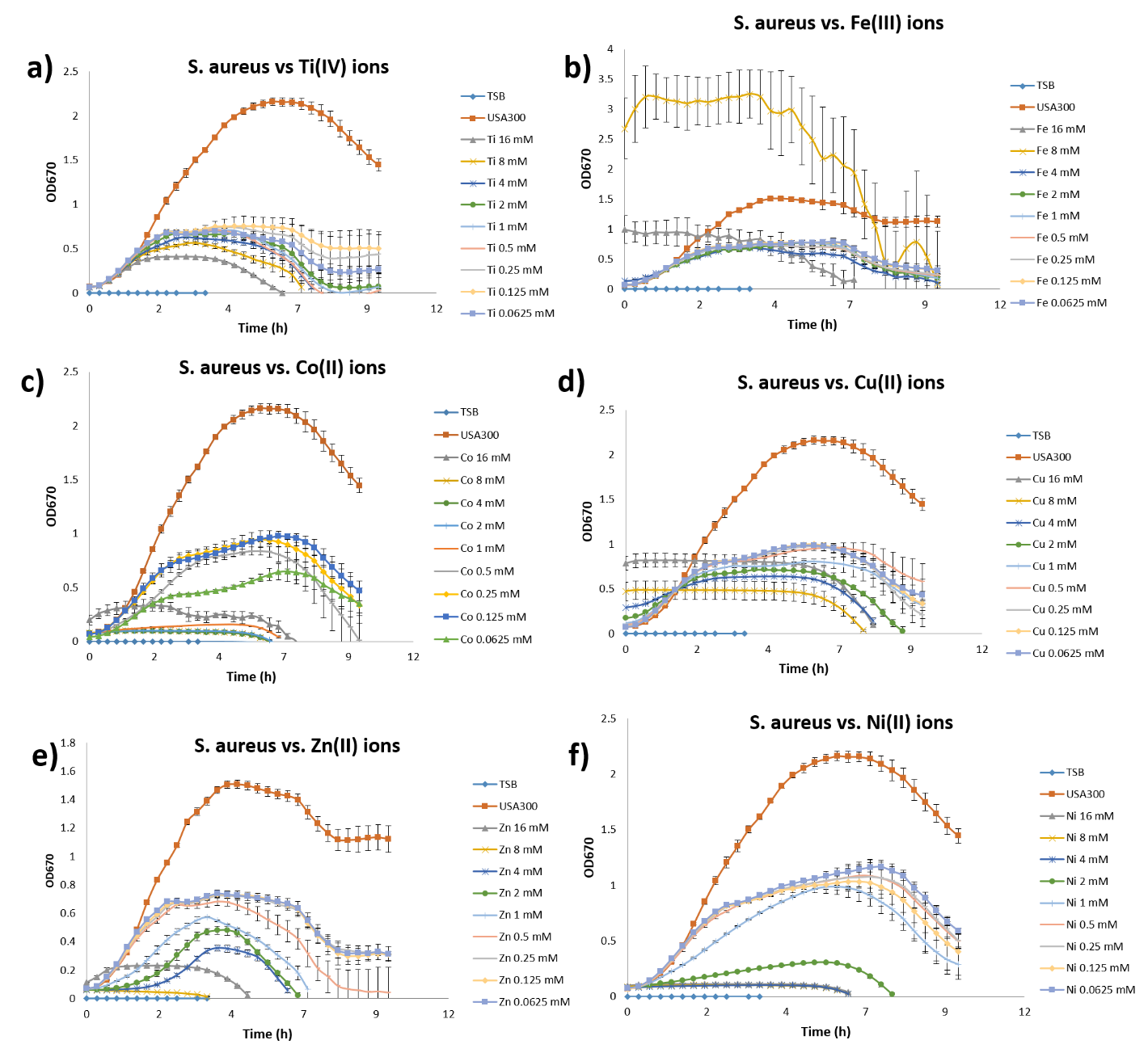


**Figure S3**. The bacterial growth kinetics of *S. aureus* with metal ions determined by UV-vis spectroscopy. The bacteria were incubated in metal ion solutions of Ti^IV^ (a), Fe^III^ (b), Co^II^ (c), Cu^II^ (d), Zn^II^ (e) and Ni^II^ (f).
